# Auxiliary Metabolic Gene Functions in Pelagic and Benthic Viruses of the Baltic Sea

**DOI:** 10.3389/fmicb.2022.863620

**Published:** 2022-07-07

**Authors:** Benedikt Heyerhoff, Bert Engelen, Carina Bunse

**Affiliations:** Institute for Chemistry and Biology of the Marine Environment, University of Oldenburg, Oldenburg, Germany

**Keywords:** bacteriophage, AMGs, salinity, marine, sediment

## Abstract

Marine microbial communities are facing various ecosystem fluctuations (e.g., temperature, organic matter concentration, salinity, or redox regimes) and thus have to be highly adaptive. This might be supported by the acquisition of auxiliary metabolic genes (AMGs) originating from virus infections. Marine bacteriophages frequently contain AMGs, which allow them to augment their host’s metabolism or enhance virus fitness. These genes encode proteins for the same metabolic functions as their highly similar host homologs. In the present study, we analyzed the diversity, distribution, and composition of marine viruses, focusing on AMGs to identify their putative ecologic role. We analyzed viruses and assemblies of 212 publicly available metagenomes obtained from sediment and water samples across the Baltic Sea. In general, the virus composition in both compartments differed compositionally. While the predominant viral lifestyle was found to be lytic, lysogeny was more prevalent in sediments than in the pelagic samples. The highest proportion of AMGs was identified in the genomes of *Myoviridae*. Overall, the most abundantly occurring AMGs are encoded for functions that protect viruses from degradation by their hosts, such as methylases. Additionally, some detected AMGs are known to be involved in photosynthesis, 7-cyano-7-deazaguanine synthesis, and cobalamin biosynthesis among other functions. Several AMGs that were identified in this study were previously detected in a large-scale analysis including metagenomes from various origins, i.e., different marine sites, wastewater, and the human gut. This supports the theory of globally conserved core AMGs that are spread over virus genomes, regardless of host or environment.

## Introduction

Viruses are the most abundant biotic entities on Earth and are ubiquitous in the marine environment. Bacteriophages, viruses that infect bacteria, occur in concentrations of up to 10^7^ viruses per ml marine surface waters, often outnumbering their hosts by 10-fold (Wommack and Colwell, 2000). Abundances of viruses in marine sediments are even higher with 10^7^-10^10^ viral particles per g of dry sediment (Danovaro et al., 2008a). With an estimated number of 10^30^ viruses in the world’s oceans (Breitbart, 2012), viruses play an important role in controlling marine bacterial populations through virus-induced mortality and represent a substantial reservoir of genetic diversity (Suttle, 2007). The exact numbers of virus-induced mortality are environment-dependent but increase with water depth and are as high as 90% at depths below 1,000 m (Danovaro et al., 2008b; Breitbart et al., 2018). Virus-induced mortality has major implications on global carbon and nutrient cycling, as it leads to a conversion of biomass to dissolved organic matter (DOM), enabling a reuptake by prokaryotes as well as preventing the transfer of DOM into higher trophic levels (Fuhrman, 1999; Wilhelm and Suttle, 1999; Suttle, 2005).

The Baltic Sea is one of the largest brackish water bodies on Earth, characterized by high rates of sedimentation (Ilus et al., 2001), high nutrient and DOC concentrations, and seasonal temperature variations of > 15°C (Bunse et al., 2019), as well as substantial riverine influx of freshwater that establishes the north-southerly salinity gradient. These mechanisms result in a stratification of the Baltic Sea and a constant halocline at a water depth of 40–80 m (Vali et al., 2013). However, the connection to the North Sea allows inflow events of saline and oxygenated water to occur irregularly (Meier et al., 2006; Reissmann et al., 2009). Prolonged stagnation further divides, e.g., deep basins of the Baltic Sea into an oxygenated layer and underlying anoxic waters, separated by the pelagic redoxcline (Labrenz et al., 2007). This distinct zonation also divides bacterial mortality factors, such as grazing and viral lysis (Pernthaler, 2005). The majority of grazing occurs in oxygenated waters, while viral lysis becomes the predominant mortality factor in anoxic layers (Weinbauer et al., 2003; Kostner et al., 2017). The functional importance of viruses in the Baltic Sea becomes apparent at the phosphorous (P)-limited Ore Estuary in the northern Baltic Sea. Here, viral lysis is supplying the dissolved DNA pool with up to 25% of its total volume. The uptake of dissolved DNA covers up to 70% of the bacterioplankton’s P-demand and thus supports their growth (Riemann et al., 2009). Stratification continues through Baltic Sea sediments, which are, like other marine sediments, vertically stratified and follow a redox gradient exhibiting decreasing free energy yield (Sørensen et al., 1979). High sedimentation rates and high organic matter concentrations hence harbor highly active bacterial communities and associated phages, even in deep subsurface sediments (Jørgensen et al., 2020). The most studied viruses of the Baltic Sea today are the bacteriophages of the Bacteroidetes phylum (Šulčius and Holmfeldt, 2016). Studies investigating other phyla such as Proteobacteria and Cyanobacteria remain scarce (Zeigler Allen et al., 2017; Nilsson et al., 2022, 2019).

A typical trait of marine bacteriophages is the ability to augment their host’s metabolism through the promotion of auxiliary metabolic genes (AMGs) (Breitbart et al., 2007; Williamson et al., 2008). Viral AMGs are genes of high similarity to host homologs. They are introduced during viral infection and encode for the same metabolic functions as those proteins of the hosts the originate from Thompson et al. (2011). AMGs were first discovered in marine heterotrophs and Cyanobacteria in the early 2000s (Rohwer et al., 2000; Mann et al., 2003; Lindell et al., 2004b). AMGs of cyanophages are associated with a variety of functions, such as energy conservation as part of Photosystem II (Mann et al., 2003; Sharon et al., 2011). Some cyanophage genomes contain over 20 AMGs that can alter the electron transport chain or enhance the carbon metabolism of their hosts (Hellweger, 2009; Sullivan et al., 2010; Thompson et al., 2011; Crummett et al., 2016). Other functions of AMGs include, e.g., the acceleration of nucleotide biosynthesis in roseophages or sulfur oxidation genes in deep-sea viruses (Anantharaman et al., 2014; Zheng et al., 2021). Through contextual distribution and maintenance of particular AMGs in the environment, viruses increase their own fitness. While most AMGs seem to affect functions of global biogeochemical cycles, genes that increase host virulence occur as well. Here, the most famous example is the filamentous CTX bacteriophage, which carries the toxin that causes the virulence of *Vibrio cholerae* (Waldor and Mekalanos, 1996). In the past, AMG identification was performed through manual inspection and functional annotation. The advance toward scalable approaches in AMG identification through new bioinformatic tools has recently allowed for large-scale assessments across whole ecosystems and has further emphasized the ecological importance of viruses (Kieft et al., 2020, 2021). The aim of the present study was to examine the diversity and composition of benthic and pelagic viral assemblies across the Baltic Sea. We focused on how salinity as an environmental driver influenced their latitudinal distribution. Therefore, we downloaded and analyzed 212 publicly available Baltic Sea metagenomes from the National Center for Biotechnology Information (NCBI) sequence read archive (SRA). We separately analyzed the viral composition and distribution in Baltic Sea sediments and the water column. In the current study, we hypothesize that virus diversity differs in both compartments due to characteristic environmental factors. We further identified AMGs within the metagenomes and analyzed their composition and distribution along the north-southerly salinity gradient of the Baltic Sea. We hypothesize that the identified AMGs enhance the fitness of the viruses and putatively support their host.

## Materials and Methods

### Metagenomic Data From the Baltic Sea

**FIGURE 1:**
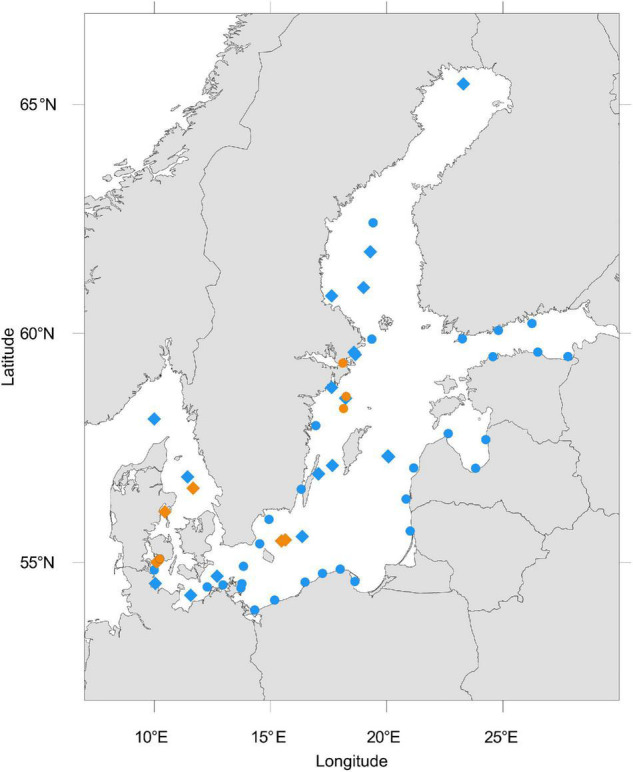
Location of analyzed Baltic Sea metagenomes downloaded from NCBI. The blue color represents metagenomes from the water column and orange represents those from sediments. Dots represent single metagenomes, diamond shapes represent a depth profile of sediment or water metagenomes, or time-series samples. Some metagenomes were sampled in close proximity to another such that overlaying symbols may entail more than one metagenome.

We retrieved a total of 212 publicly available metagenomes from sequence-based metagenomic studies of Baltic Sea sediments and water samples (Figure 1). The included data result from the project IDs PRJEB22997 (Alneberg et al., 2018), PRJEB34883 (Alneberg et al., 2020), PRJEB6616 (Thureborn et al., 2016), PRJEB8682 (Kopf et al., 2015), PRJNA308531 (Andren et al., 2015), PRJNA273799 (Hugerth et al., 2015), PRJNA297401, PRJNA322246 (Asplund-Samuelsson et al., 2016), PRJNA367442, PRJNA337783, PRJNA433242 (Zinke et al., 2017), and PRJNA337783 (Espínola et al., 2018), which were obtained from the NCBI SRA (accessed May–June 2020). The data originate from different sample sets that comprise different filter fractions, community members, and environments and likely also differ in sampling method as well as DNA extraction. The location of all metagenomic samples analyzed within this study were plotted using the R package oceanmaps (Bauer, 2020). All metadata and published environmental data available from these projects are summarized in Supplementary Table 1.

### Sequence Quality Analysis and Assembly

Sequence quality control analysis was performed using FastQC (Andrews, 2010). Metagenomic read files were then trimmed of adapters using BBDuk (Bushnell, 2018). Quality trimming was also performed using BBDuk with the quality threshold set to Q30. High-quality metagenomes were assembled using MEGAHIT (Li et al., 2015) with the meta-large flag, as suggested for complex metagenomes. Statistics about the quality of assembled metagenomes were analyzed using MetaQUAST v5.0.2 (Mikheenko et al., 2016), Supplementary Table 2.

### Identification of Viral Contigs

The *in silico* prediction of phage scaffolds and viral AMGs was done on all 212 metagenomes using VIBRANT v1.2.1 (Kieft et al., 2020) with default settings. Vibrant can accurately recover viruses and AMGs by applying machine learning and a protein similarity approach. The quality of contigs identified by VIBRANT was further assessed with CheckV (Nayfach et al., 2021) retaining contigs > 3 kb and filtering any non-viral contigs. Abundance profiles of AMGs identified by VIBRANT were generated by mapping quality-controlled metagenome reads to the AMGs using Bowtie2 (v1.2.2) (Langmead and Salzberg, 2012). The sequence mapping files were handled and converted using SAMtools (v1.9-58) (Li et al., 2009). Fragments Per Kilobase per Million (FPKM) mapped reads were calculated as the number of mapped reads times 10^9^ divided by the total number of mapped reads per sample multiplied by the gene length. Viral taxonomy of AMGs located on filtered contigs was assigned using DIAMOND BLASTp v0.9.30 (*E*-value of < 0.0001, bit score ≥ 50) and the “—very-sensitive” preset (Buchfink et al., 2015). Viral hosts of identified contigs were assigned with VirHostMatcher-Net using default settings (Wang et al., 2020).

To gain broader context over the identified viral contigs, they were compared to viral contigs in the following public databases: (1) Global oceans virome (GOV) 2.0 Seawater (Gregory et al., 2019) and (2) Stordalen thawing permafrost (Emerson et al., 2018). Open reading frames for each viral contig were called using Prodigal V2.6.3 (Hyatt et al., 2010). Predicted protein sequences were used as input from vConTACT2 (Bin Jang et al., 2019). Viral Refseq (211) was used as a reference database (O’Leary et al., 2016). Diamond BLASTp was used for the protein-protein similarity method. All other parameters were set as default. The gene network was visualized using Cytoscape v3.9.1 (Shannon et al., 2003).

Inferring viral taxonomy through clusters identified by vConTACT2 resulted in small numbers of contigs that could be taxonomically assigned. Thus, to gain an overview of viral families present in Baltic Sea metagenomes, we used Kraken 2 to assign viral taxonomy from filtered high-quality unassembled reads applying default settings and using viral sequences from the NCBI non-redundant nucleotide database (release 211) as reference (O’Leary et al., 2016; Wood et al., 2019). Kraken 2 infers taxonomic classification by using exact k-mer matching and assigning query sequences to the lowest common ancestor. Accurate species abundance re-estimation was calculated using Bayesian Reestimation of Abundance with KrakEN (Bracken) with default settings for all metagenomes (Lu et al., 2017).

### Statistical Analysis

Data processing and visualization were carried out with R version 4.0.5 (R Core Team, 2021) and the tidyverse package (Wickham et al., 2019). Bray–Curtis distances of relative viral abundances at each station were visualized by non-metric multidimensional scaling (NMDS) (*k* = 2; 999 permutations) using the vegan (v2.5-7) package (Oksanen et al., 2013). The top nine most abundant virus families were fitted to the ordination using the vegan envfit-function with 999 permutations and removal of unavailable data enabled. Salinity isobars were added using the ordiplot function. Alpha diversity was calculated with the Shannon diversity index and centered log-ratio normalized counts using the phyloseq package in R (McMurdie and Holmes, 2013; Gloor et al., 2017). A Wilcoxon test was conducted to test the significant difference in alpha diversity between the viral composition of water and sediment metagenomes using the Wilcox test R function. Beta diversity was analyzed by using the Aitchinson distance by applying principal component analysis (PCA) to the centered log-ratio transformed counts. Zero counts were avoided by adding a pseudo count to avoid errors during clr transformation. Differential abundance testing was done using DESeq2 normalized counts (Love et al., 2014). A permutational multivariate analysis of variance (PERMANOVA; function Adonis, method = “Euclidean,” Permutations = 999) was done to test if beta diversity was significantly different in water or sediment metagenomes. The 20 most differentially abundant taxa with the smallest p.adj values (*p.adj* < *0.001*) were plotted in a heatmap using the ComplexHeatmap package, using the dendextend R package for hierarchical clustering analysis (Galili, 2015; Gu et al., 2016).

## Results

### The Viral Composition Differs Between Baltic Sea Sediments and Water Column Samples

In this study, we assembled and analyzed 212 publicly available metagenomes from Baltic Sea sediments and water column samples. The metagenomes were constructed from samples collected between 53°N and 65°N latitudes in the years 2008–2015. In total, 37 of the analyzed metagenomes originated from sediments and 175 from the water column samples. We identified 102,892 viral contigs > 3 kb after quality filtering with CheckV of which 7,540 contained at least one AMG (Supplementary Table 3).

**FIGURE 2:**
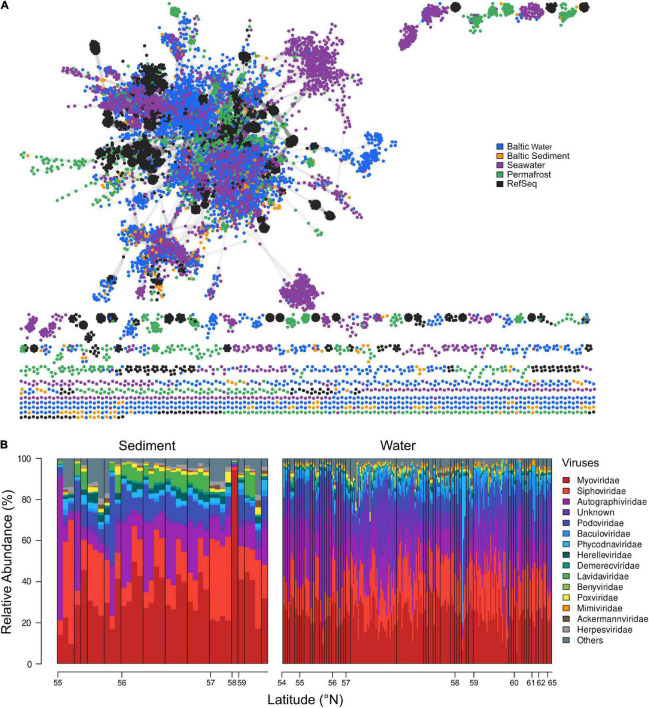
**(A)** Gene-sharing network of viral sequence space based upon assembled viral genomes from Baltic Sea sediment and water column, GOV seawater, permafrost, and viral RefSeq genomes. **(B)** Relative abundance (%) and distribution of the top 15 most abundant virus families, ordered in a south-northerly arrangement by latitude (°N). Viral families are plotted in an increasing order across all water and sediment stations. The remaining families are summarized in “Others.” Black bars delimit depth profiles and are ordered by increasing sediment or water depth, respectively.

We investigated the relationship of Baltic Sea viral contigs, with other publicly available viral sequences from different ecosystems (Figure 2A). Baltic Sea sediment and water column viral contigs as well as viral contigs from permafrost, GOV seawater, and RefSeq were grouped into 2,638 clusters (Supplementary Table 4). Viral contigs originating from the Baltic Sea water column overlapped relatively well with GOV seawater viruses; however, some small outliers occurred with the largest one displayed on the right side of the network graph (Figure 2A). The separate cluster of water column viruses was exclusively lytic but appeared throughout the Baltic Sea from 53° N to 65° N from surface water to 241.7 m water depth in the Skagerrak and seemed not to be impacted by salinity or temperature. None of the clusters contained vOTUs of all analyzed ecosystems. Rather, the Baltic Sea water column shared 35 clusters with GOV seawater and 11 clusters with Baltic Sea sediments but only 5 clusters contained vOTUs from Baltic Sea sediments, water column, and GOV seawater. Baltic Sea sediment shared 4 clusters with Stordalen permafrost and only very few vOTUs (0.4%) clustered with taxonomically known genomes from viral RefSeq. The limited number of clusters between viral genomes of analyzed ecosystems may reflect the high habitat specificity of viruses. The limited number of taxonomically identifiable viral genomes led us to use another method of taxonomic identification of viruses *via* Kraken 2 and allowed for a more detailed look at present viral families.

The Baltic Sea viral composition was versatile and locally differentiated. The most evenly distributed viruses in analyzed water and sediment samples were *Myoviridae*, showing abundances of around 20–40% (Figure 2B). One outlier was observed at 58°N, where they comprised 83.98% of the total viral composition. In sediments, the lowest abundance of *Myoviridae* occurred at around 55°N. *Siphoviridae* represented the second most abundant viral family and occurred in a less evenly distributed manner than *Myoviridae*. They dominated the sediment viral communities between 55°N and 56°N and between 57° and 58°N, where they made up to 55% of the viral assemblies. In the water column, a larger fraction of unknown and *Phycodnaviridae* viral families were found compared to sediments, of which the latter accounted for more than 80% of some communities in the water column. Overall, we did not observe a major trend along the latitudinal gradient.

**FIGURE 3:**
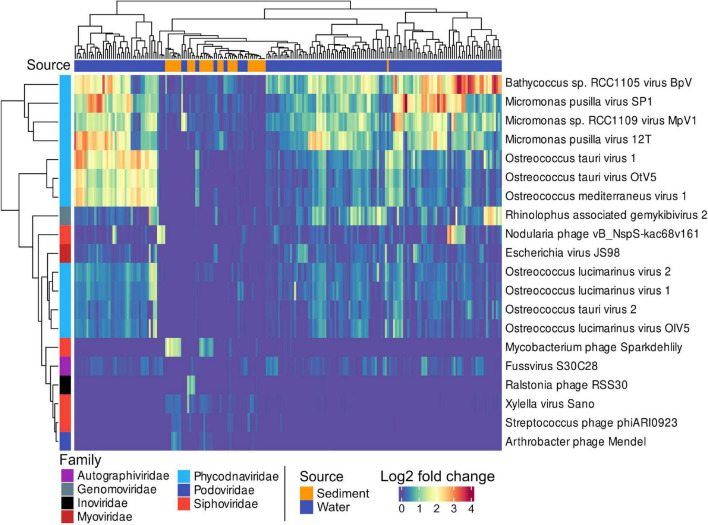
A heatmap showing the 20 most significant, differentially abundant viral taxa between sediments and water samples. The Matrix was DESeq2 normalized showing the 20 most differentially abundant taxa with the smallest *p*.adj values (< *0.0001*). Sources of samples are indicated by blue or orange in the top color bar, and viral families by the color bar on the left side.

Members of the *Phycodnaviridae* family were the most differentially abundant viruses and distinguished pelagic from benthic viral assemblies. *Phycodnaviridae* infect *Bathycoccus, Micromonas*, and *Ostreococcus* genera, which belong to the green algae (Figure 3). Sediment stations from the Bornholm Basin and the Bay of Aarhus (SRR3081534, SRR3085416, SRR3085435, SRR3085585, SRR3089827, SRR3091743, SRR3095933, SRR3095939, and SRR7067081) sampled at depths of 0.75–3 m below sea floor (mbsf) displayed higher counts of the differentially abundant Mycobacterium phage Sparkdehlily. Deep subsurface stations (SRR12059190, SRR12059191, and SRR12059199) sampled at 24.1, 24.1, and 67.5 mbsf, close to the island of Anholt and the Little Belt, were defined by the differentially abundant Ralstonia Phage RSS30.

### Viruses in Sediments and Water Column are Similarly Diverse

**FIGURE 4:**
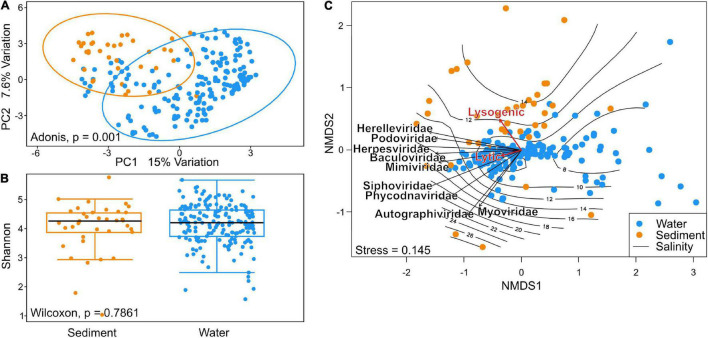
**(A)** Principal component analysis of beta diversity using Aitchinson distance shows distinct patterns in community composition of sediment and water communities. *(B)* Shannon alpha diversity of Baltic Sea viral communities in Wilcoxon *p*-value of 0.7861 indicates no relevant difference in per-sample diversity when comparing species richness of sediment and water communities. **(C)** Non-metric multidimensional scaling (NMDS) analysis using Bray–Curtis dissimilarity. Vectors of the top nine most abundant families and of environmental factors were fitted to the ordination using the envfit function. A separation of sediment and water stations can be observed along the salinity gradient plotted using the ordisurf function. Envfit vectors indicate a tendency of viruses toward the lysogenic lifestyle in sediment metagenomes and lytic lifestyle in the water columns.

Beta diversity of viral communities revealed a distinct pattern in virus composition between sediment and water viral assemblies, explaining 22.6% of total variation (Adonis, *p* = 0.001). The two plotted components separated viral taxa from sediments and water samples distinctively, with a small remaining overlap (Figure 4A). The median viral alpha diversities (Shannon index) of the sediment stations were 4.25, and 4.2 for water stations, indicating no statistically significant difference calculated by the Wilcoxon test (*p*-value = 0.7861) (Figure 4B). Due to the compositional nature of the metagenomic data used in this study, we assessed a possible batch effect *via* Bray–Curtis distance and visualized the results in an NMDS ordination (Supplementary Figure 3). We additionally conducted a PERMANOVA to test whether the different sequencing projects caused a batch effect within our NMDS ordinations. While some project-specific clustering could be observed within the ordination, the PERMANOVA showed these effects to be less important (*R*^2^ = 0.30723, *P* < 0.001) (Supplementary Figure 3). The batch effect among just water and sediment stations was also not relevant (*R*^2^ = 0.23696, *p* < 0.001 and *R*^2^ = 0.28178, *p* < 0.001).

Viral community dissimilarity was investigated using non-metric multidimensional scaling (NMDS) analysis (Figure 4C). Samples close to the center of the ordination represented samples with similar viral compositions. Sediment samples spread more throughout the ordination and appeared more toward higher salinity, while water stations clustered closer together around the center of the ordination and around the 10 PSU isobar. The top nine most abundant viral families were plotted into the ordination. Among these, *Autographiviridae* and *Myoviridae* aligned more with the *y*-axis, whereas the other viral families aligned more with the cluster of samples that aligned with the *x*-axis in the lower left quadrant. The *Siphoviridae* and *Phycodnaviridae* vectors were located somewhat separately from other viral families. While the lytic lifestyle aligned more with water stations, the lysogenic lifestyle appeared more in the sediments. However, the lytic lifestyle was found to be the overall dominant viral lifestyle in the Baltic Sea as shown in Supplementary Figure 1.

### Viruses From Water and Sediment Carry Auxiliary Metabolic Genes Specific to the Environment

**FIGURE 5:**
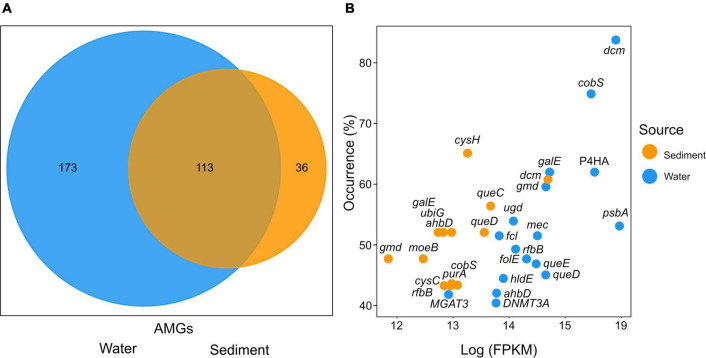
**(A)** Number of unique KEGG orthologs specific to either water or sediment viral composition. The overlap displays the number of unique KEGG orthologs found in both sediments and the water column. **(B)** The distribution of the most abundant KEGG orthologs plotted by station occurrence over Fragments Per Kilobase per Million reads (FPKM) assigned. The x-axis displays the log FPKM count of KEGG orthologs. The *y*-axis indicates the occurrence in stations (%) of samples in which the KEGG orthologs were found calculated per source. The cutoff was chosen at 40% of stations. Colors indicate the gene origin.

In both, the sediment and water metagenome AMGs could be assigned to 322 unique KEGG orthologs (Supplementary Table 5). The water column was the more diverse habitat with 173 unique KEGG orthologs assigned in total. While 36 unique KEGG orthologs could be identified in sediments, 113 identified AMGs were found in both habitats (Figure 5A). In the water column, AMGs of unknown function accounted for 13.1% of all mappable FPKM and 15.2% in sediments.

The most abundant AMGs are visualized by the percent occurrence of stations over their log sum of FPKM (Figure 5B and Table 1). Six outliers stand out among the AMGs identified in the water column: *dcm, cobS, gale, P4HA, gmd*, and *psbA.* The two most abundantly occurring AMGs in this study were encoding for *dcm/DNMT1* and *cobS* occurring in 84 and 75% of stations, while *gale, P4HA, gmd*, and *psbA* occurred in 62, 62, 60, and 54% of water respectively. In contrast, the most abundant outliers of sediment AMGs, *cysH, dcm*, and *queC* occurred in 65, 61, and 57% of stations, respectively. These genes encode for the phosphoadenosine phosphosulfate reductase, DNA (cytosine-5)-methyltransferase 1, and the 7-cyano-7-deazaguanine synthase.

**TABLE 1:** Most abundant KEGG orthologs with assigned metabolic pathway, the sum of assigned FPKM, and occurrence (% samples) in which the KEGG orthologs were found.

Gene name	KEGG ortholog	Log (FPKM)	Station occurrence (%)
**Sediment**			
*cysH*	K00390	12.85	65.2
*DNMT1, dcm*	K00558	14.29	60.9
*queC*	K06920	13.27	56.5
*ubiG*	K00568	12.42	52.2
*queD*	K01737	13.15	52.2
*galE*	K01784	12.33	52.2
*ahbD*	K22227	12.57	52.2
**Water**			
DNMT1, *dcm*	K00558	15.50	83.9
*cobS*	K09882	15.05	75.0
*P4HA*	K00472	15.12	62.1
*galE*	K01784	14.32	62.1
*gmd*	K01711	14.25	59.7
*ugd*	K00012	13.67	54.0
*psbA*	K02703	15.56	53.2
*fcl*	K02377	13.42	51.6
*mec*	K21140	14.10	51.6

### Auxiliary Metabolic Gene Distribution Along the Salinity Gradient of the Baltic Sea

**FIGURE 6:**
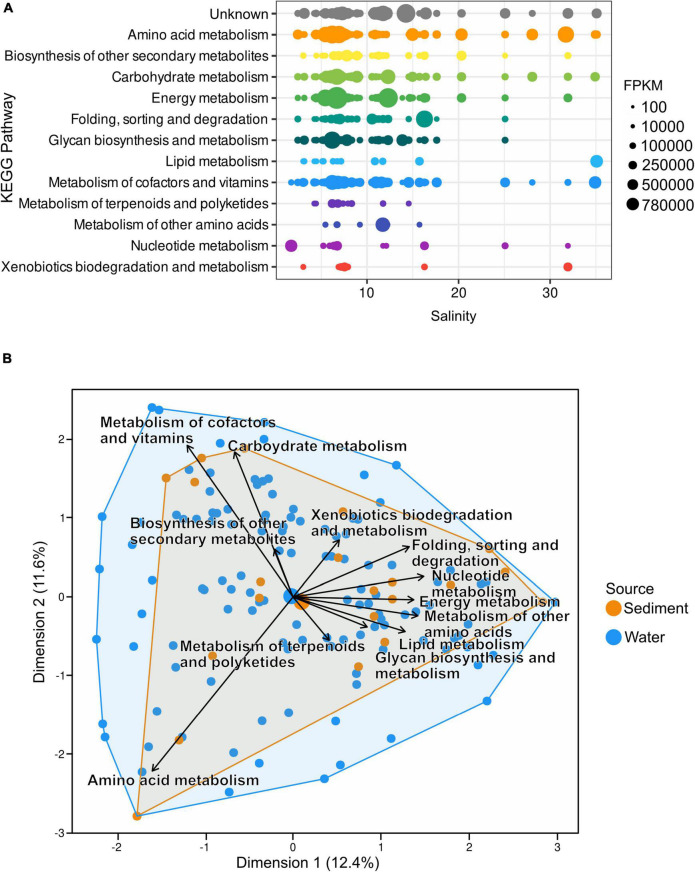
**(A)** Distribution of AMGs along the salinity gradient of the Baltic Sea; both water and sediment samples are depicted together. The number of AMGs was normalized to AMG per million reads. **(B)** Principal component analysis of Hellinger transformed AMG counts shows most samples cluster close to the center of the ordination. However, vectors of metabolic pathways indicate no uniform presence of metabolic pathways at all stations.

While the most predominant salinity gradient stretches from the south to the north, differences in salinity also occur by depth due to the higher density of saline water. “Amino acid” and “carbohydrate metabolism” AMGs as well as “Metabolism of cofactors and vitamins” appeared most consistently along the salinity gradient. The highest number of fragments were assigned to the “Energy metabolism” at salinities of 6.7, 6.71, and 12.3. However, while “Metabolism of cofactors and vitamins” did not occur in high numbers at certain salinities, 23.2% of all assigned FPKM were assigned to this pathway as it appears most consistently along the salinity gradient. It is closely followed by “Amino acid metabolism” with 18.1% and “Energy metabolism” and unknown pathways with 14.9 and 14.1%, respectively (Figure 6A and Supplementary Table 6). PCA of Hellinger transformed AMG counts clustered most of the analyzed metagenomic samples evenly throughout the ordination (Figure 6B). While most pathway vectors cluster relatively closely together, “Amino acid metabolism”, “Metabolism of cofactors and vitamins”, and “Carbohydrate metabolism” are separated from the other vectors.

### Cyanobacteria and Proteobacteria Are the Most Abundant Prokaryotic Hosts

**FIGURE 7:**
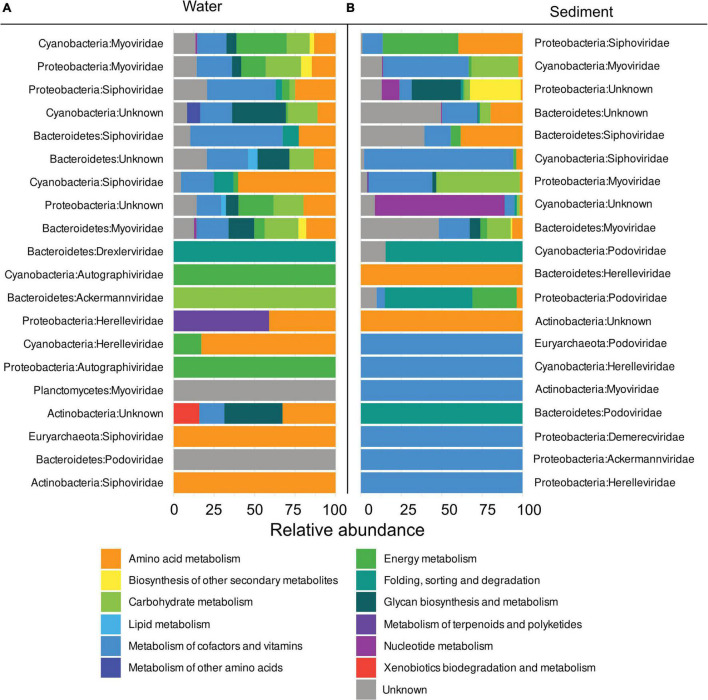
Relative abundance of metabolic pathways per host phylum and viral family in descending order by the sum of total FPKM assigned to metabolic pathways in **(A)** the water columns and **(B)** sediments.

Prokaryotic hosts of AMGs were assigned with the VirHostMatcher-Net tool, allowing the identification of hosts affected most by viral metabolic interference. In the water column, *Cyanobacteria* were found to be the hosts with the highest number of assigned FPKM with 43.9% of all FPKM assigned to them. *Proteobacteria* and *Bacteroidetes* followed closely with 34 and 20.2% of total assigned FPKM respectively. In sediments, most FPKM were assigned to *Proteobacteria* (53.5%), Cyanobacteria (24.1%), and Bacteroidetes (21.9%) (Figures 7A,B and Supplementary Table 7).

### Most FPKM Assigned to Auxiliary Metabolic Genes of Myo- and Siphoviridae

In the water column, 48.8% of FPKM were assigned to AMGs identified in the *Myoviridae* family and 21.7% in the *Siphoviridae* family. While some FPKM were also assigned to AMGs of other viral families (i.e., *Herelleviridae* and *Ackermannviridae*), they only accounted for 1.9 and 0.6%, respectively (Figure 7A). In the sediment, 40.5% of FPKM could be assigned to *Siphoviridae* and 23% to *Myoviridae*, while *Podoviridae* and *Herelleviridae* only accounted for 1.88 and 0.31%, respectively (Supplementary Table 7).

*Myoviridae* procured the most diverse pathways. AMGs assigned to this family were found in nine out of twelve KEGG pathways that are detected in all metagenomes. The second most versatile family of viruses was *Siphoviridae*, which carried AMGs belonging to 8 out of 12 of all KEGG pathways, while *Siphoviridae* carried AMGs from 7 out of 12 KEGG hierarchies. Notably, more AMGs of the “Metabolism of cofactors and vitamins” pathways were present in *Myoviridae* in sediments, whereas AMGs of “Biosynthesis of other secondary metabolites” and “Energy metabolism” in the water columns were more pronounced compared to sediments. AMGs assigned to the “Amino acid metabolism” and “Metabolism of cofactors and vitamins” were the most abundantly occurring type of AMGs that were present in the top four most abundant viral families, which made up 98% of all assigned AMGs.

## Discussion

### Diverse Viral Composition in Sediments and the Water Column

In general, *Myoviridae* and *Siphoviridae* were detected as the most abundant viral families within the whole metagenomic data set. However, while some overlaps occur, the viral composition of sediment and water stations clustered separately from each other. Even though the viral assembly in sediments and the water columns were similar in species richness, they differed in beta diversity. Minor differences in viral diversity of the samples can be expected due to varying sampling methods and filter fractions used in the individual projects, that are summarized in this meta-analysis. Specifically, differences in the viral composition of sediments and the water column were defined through the differentially abundant *Phycodnaviridae*, which contributed up to 80% of total relative abundance at the surface. While in compositional datasets, relative abundances cannot be used to infer absolute viral absolute viral particles, cell counts, or gene abundances, we noted decreasing relative abundances of *Phycodnaviridae* with decreasing water depth. The high relative abundances of *Phycodnaviridae* in the subsurface sediments at 10.1 mbsf are likely the result of high sedimentation rates in the Baltic Sea and a lack of benthic animals due to limited oxygen supply, allowing the undisturbed formation of sediment layers. While most sediment stations in our study were similar to each other, small increases of Ralstonia phage RSS30 were observed in subsurface stations sampled from Aarhus Bay sediments, Denmark. Cyanobacteria in our study were abundant hosts, yet the identification of *Prochloraceae* as hosts likely resulted from a database bias, as previous studies have not detected them in the Baltic Sea (Bertos-Fortis et al., 2016; Celepli et al., 2017). This interpretation is supported by Celepli et al. (2017), who have generated hits of *Prochloraceae* in their metagenomic data set, while their 16S rRNA analysis also revealed the absence of *Prochloraceae*. Other unicellular Cyanobacteria (*Synechococcus* and *Cyanobium*) are frequently found in datasets of the Baltic Sea and likely contributed as cyanophage hosts also in these metagenomes (Haverkamp et al., 2009; Larsson et al., 2014; Broman et al., 2021).

### The Lytic Lifestyle Prevails in the Baltic Sea

In the past, viruses have mostly been studied under laboratory conditions with a focus on three life cycles: chronic, lytic, and lysogenic. During the chronic lifestyle, phages enter a productive replication cycle, releasing virions without lysing their host. Exhibiting the lytic lifestyle, viruses lyse their hosts upon infection, releasing viral progeny into the environment (Correa et al., 2021). In coastal waters, Wilcox and Fuhrman (1994) reported the lytic lifestyle to be the most abundant. During the lysogenic cycle, a non-productive infection occurs by integrating the virus into the host’s genome and replicating it along with the host. A virus may exit the lysogenic cycle and become lytic through specific factors or by spontaneous switching of lifestyles. Previous laboratory studies have looked at abiotic factors, where life lifestyle switching was influenced by phosphate availability or salinity (Wilson et al., 1996; McDaniel et al., 2002; Bettarel et al., 2011).

However, while lytic, lysogenic, and chronic lifestyles are reflective of viral behavior in the laboratory, they are not entirely representative of natural behavior. Instead, viral lifestyles are controlled by complex interactions and represent a continuum rather than infection categories (Weitz et al., 2019; Correa et al., 2021). External mechanisms such as diel and seasonal changes may influence viral lifestyles (Ballaud et al., 2016; Brum et al., 2016; Puxty et al., 2018). However, recent studies postulate that switching between lysogeny and lysis is especially influenced by host density (Erez et al., 2017). In the environment, lysogeny has been found to be a low-density refugium occurring at low host abundance. The refugium theory assumes exponentially growing communities to be rich in intracellular energy which favors lysis, whereas communities of low abundances are depleted of intracellular energy sources, which favors lysogeny (Erez et al., 2017). Yet, lysogeny has been found to be a survival strategy in low and high host-density conditions (Mizuno et al., 2016; Kim and Bae, 2018; Coutinho et al., 2019; Luo et al., 2020).

Lysogeny, as a result of high host density, has been described as the “piggyback-the-winner” model (Knowles et al., 2016). Additionally, the “killing the winner” theory predicts that hosts of the highest density are lysed (Thingstad, 2000). Low density and energy availability favor lysogeny, but increasing host density facilitates induction and lysis as denser communities administer more internal energy. Lysogeny continues to decrease until host densities of ∼10^6^ cells ml^–1^ are reached, which are typically observed in the open oceans (Luque and Silveira, 2020). Higher densities increase the chances of coinfections with other viruses. Thus, lysogeny becomes a favorable lifestyle (Luque and Silveira, 2020). This switching might be communicated among phages as observed by Erez et al. (2017). Here, the *Bacillus* infecting phages of the SPbeta group released small peptides into the medium, signaling switching to the lysogenic lifestyle at higher concentrations of the respective compounds. This mechanism has been identified in different phages, each of which utilized different versions of the communication peptide (Erez et al., 2017).

Considering the abovementioned complexity of viral lifestyles, the observed dominance of identified lytic viral contigs in the Baltic Sea (Supplementary Figure 1) provides just a snapshot derived from genomic sequences of complex and dynamic systems. For instance, the metagenomic samples which are the basis of this study were taken at different time points and locations, thus allowing only remarks about the moment of sampling. Furthermore, the methodological limitation of the VIBRANT tool used to classify contigs as lytic or lysogenic has to be considered. VIBRANT assigns the lysogenic lifestyle by using surrounding host genome elements or integrases as evidence, limiting the identification of lysogenic contigs. The detected viral contigs might be lysogenic but the absence of the aforementioned properties in partial genomes could lead VIBRANT to falsely categorize them as lytic rather than lysogenic.

### Auxiliary Metabolic Genes Catalyze Virus-Host Interactions

Viruses utilize AMGs to alter their host’s rate-limited cellular processes during infection (Sullivan et al., 2006). The roles of such AMGs are not random but critical for the successful proliferation of the viruses. Here, the most abundantly distributed AMG was the *dcm* gene, encoding for a methyltransferase. These enzymes are ubiquitously found in prokaryotes and are often associated with cognate restriction endonucleases, forming a restriction-modification system that protects bacterial cells from foreign DNA invasion. In bacteriophages, the so-called orphan methyltransferases appear without these endonucleases and are involved in regulatory activities to protect the phage DNA from being digested (Schlagman et al., 1986; Boye and Løbner-Olesen, 1990; Palmer and Marinus, 1994; Kossykh et al., 1995). While methylation is a well-known way of escaping host restriction in viruses, they also procured other means of nucleotide modifications. The genes *folE, queD, queE*, and *queC*, are necessary for 7-cyano-7-deazaguanine (preQ_0_) synthesis. These genes were among the most abundantly occurring AMGs in our dataset with the genes *queD* and *queC* both occurring in 52% of sediment and *queE* in 46% of water samples. This indicates that preQ_0_ is important in viral replication. Queuosine is a hypermodified guanosine found in tRNAs specific for four amino acids (Asp, Asn, His, Tyr) and increases translation efficiency (Sabri et al., 2011; El Yacoubi et al., 2012). The presence of preQ_0_ synthesis genes in viruses has been reported previously and protects the virus from host restriction enzymes (Hutinet et al., 2019).

Photosynthesis genes, such as the *cobS* gene among the *psbA* gene, are considered core AMGs in cyanophages (Ignacio-Espinoza and Sullivan, 2012). In our study, these occurred especially abundantly in water samples. The *cobS* gene encodes for a protein that catalyzes the final step in bacterial cobalamin (vitamin B12) biosynthesis (Magnusdottir et al., 2015). Speculations about the involvement of viruses in the cobalamin biosynthesis in the pelagic ecosystem are tempting, yet more targeted analyses and experimental evidence would be needed for a conclusive answer. The *psbA* gene is among the best-studied AMGs. It encodes for the photosystem II protein D1. Together with photosystem II protein D2, it forms a heterodimer and binds P680, which is a specific chlorophyll a and the primary electron donor of photosystem II. Marine picocyanobacteria, such as those of the genus *Synechococcus*, are among the most abundant photosynthetic organisms on Earth and are responsible for the fixation of approximately 25% of the carbon in the marine environment (Scanlan et al., 2009; Flombaum et al., 2013). Viral production in *Cyanobacteria* is limited by the availability of energy for protein synthesis during late infection. Cyanophage production correlates with irradiation intensity and is inhibited by darkness (Puxty et al., 2016; Thompson et al., 2016). To circumvent energy limitations, cyanophages augment their hosts’ metabolism by introducing genes for the photosynthetic light reactions. In the early stage of infection, CO_2_ fixation can be actively inhibited by the phages, diverting the hosts’ metabolism toward the pentose phosphate pathway, thus increasing NADPH and ribose-5-phosphate production, facilitating viral protein and DNA synthesis rather than increasing photosynthetic activity (Thompson et al., 2011). The regulatory ability of cyanophages on global carbon cycling and primary production through lysis and active augmentation of carbon fixation rates implies the importance of these phages. The AdoMet-dependent heme synthase *ahbD* is involved in protoheme biosynthesis by catalyzing the conversion of Fe-coproporphyrin III into heme. This has been studied in sulfate-reducing bacteria of the *Desulfivibrio* genus and in methanogenic Archaea (Buchenau et al., 2006). Heme is an essential prosthetic group and, among other biological processes such as respiration, is very important in photosynthesis (Layer et al., 2010). Procuring such genes could increase the energy metabolism and speed up virus production by reducing the latent period (Mann et al., 2003; Lindell et al., 2004a; Millard et al., 2004). However, while we found 261 instances of the *ahbD* AMG, only three contigs contained both, the radical SAM domain PF13186 and radical SAM domain PF04055, and were not classified as Archaea nor of *Desulfovibrio.* Hence, inferences about the function of this AMG are rather speculative. While the *ahbD* gene requires further investigation, the genes *psbA* and *cobS* highlight the importance and distribution of AMGs involved in photosynthesis in pelagic phages, emphasized also by high cyanobacteria abundances in the Baltic Sea.

The *ubiG, galE*, and *P4HA* genes cannot easily be assigned to greater metabolic functions such as photosynthesis but appear to be of similar importance. The *ubiG* gene encoding for the last step in the pathway of ubiquinone biosynthesis likely provides the phages with the ability to affect the electron transport chain. The *galE* gene encoding the UDP-glucose 4-epimerase placed third among the most abundant AMGs in the water column. It mediates the conversion of UDP-galactose and UDP-glucose in galactose metabolism (Thoden and Holden, 1998). Thus, the introduction of *galE* likely allows the virus to participate in the carbohydrate metabolism to generate energy. The similarly abundant *P4HA* gene, encoding for the prolyl 4-hydroxylase, catalyzes the hydroxylation of proline residues in peptide linkages in collagens, forming 4-hydroxyproline (Myllyharju, 2003). In viruses, collagen can be part of the tail fibers and was first detected in Paramecium bursaria Chlorella virus-1 (Eriksson et al., 1999; Rasmussen et al., 2003). In which way viruses utilize prolyl 4-hydroxylase remains cryptic. However, biological consequences of prolyl hydroxylation include altering protein conformation and protein–protein interactions but also contributing to collagen-helix stability in general (Rao and Adams, 1979).

Analogous to water samples in our study, nucleotide modification through the *dcm* and *queC* gene are the most important functions provided by viruses. As photosynthesis is less relevant in sediments, especially those of greater depths, other functions prevail. Here, the most abundantly occurring AMG not involved in DNA modification is the *cysH* gene, encoding for the phosphoadenosine 5′-phosphosulfate reductase. It was found in 65% of all sediment stations but also in 39,5% of the water stations. The presence of the *cysH* gene suggests the viral involvement in sulfur cycling, especially in viruses found in Baltic Sea sediments. The enzyme is involved in the synthesis of sulfite from phosphoadenosine 5’-phosphosulfate (PAPS) and thus part of the sulfate reduction pathway (Bick et al., 2000). The *cysH* gene has been identified in phages infecting members of the SAR11 clade, which lack the phosphoadenosine 5’-phosphosulfate reductase and other genes required in assimilatory sulfate reduction but has recently also been found to be generally widespread in marine phages (Du et al., 2021; Kieft et al., 2021).

### Conserved Core Auxiliary Metabolic Genes

Recently, Kieft et al. (2020) identified a set of AMGs found in highly different viral assemblies from various origins, i.e., human gut, marine sediment, deep subsurface, and others. Their set of globally conserved AMGs consisted of the *dcm, cysH, folE, phnP, ubiG, ubiE, waaF, moeB, ahbD, cobS, mec, queE, queD, queC* genes and occurred in at least 10 out of 12 of their studied samples. These genes were also identified in the Baltic Sea, though not all of them are among the most abundant. However, the genes *dcm, cysH, folE, cobS, mec, queE, queD, queC* are concurrent with the findings of Kieft et al. (2020) and suggest the existence of a globally distributed set of conserved core AMGs, which are present regardless of host and environment. Locally, the core AMGs might then be extended by genes specific to an environment of interest such as genes for photosynthesis. A definition of core AMGs is difficult, as setting a threshold for their occurrence at a given station or environment would be arbitrary. However, the similarity in composition of most abundantly occurring AMGs in the Baltic Sea and other environments is striking.

## Conclusion

The metagenomic analysis revealed a predominantly lytic viral life mode in the Baltic Sea, possibly aided by high nutrient availabilities and increasing lysogeny traits in sediments. We did not find major virus community differences along the north-southerly salinity gradient of the Baltic Sea. Yet, the composition of pelagic and benthic virus assemblies differed, especially in the relative abundances of *Phycodnaviridae*. Also, the functional virus AMGs differed between the pelagic and benthic samples. While viruses from the water columns procured AMGs specific for photosynthesis, viruses in sediments acquired AMGs that are part of the nutrient cycling pathways such as sulfur cycling. Other AMGs that exclusively occurred in sediments or the water column were found in low abundances and are likely linked to functions that specifically increase virus fitness in the respective ecosystem. Viruses use AMGs to evade host restriction mechanisms, i.e., by modifying their DNA through methylation or utilization of preQ_0_. These DNA modification AMGs were highly abundant in the Baltic Sea and have also been observed to be globally conserved. Our findings, therefore, strengthen the hypothesis on the existence of global core AMGs that are central to viral replication, regardless of environment and host.

## Data Availability Statement

The original contributions presented in this study are included in the article/Supplementary Material, further inquiries can be directed to the corresponding author.

## Author Contributions

BH performed the conceptualization, datamining, bioinformatics, and analyses. BE secured the funding. BH and BE wrote the manuscript. CB helped conceptualize the study and helped with data evaluation. All authors read and approved the manuscript.

## Conflict of Interest

The authors declare that the research was conducted in the absence of any commercial or financial relationships that could be construed as a potential conflict of interest.

## Publisher’s Note

All claims expressed in this article are solely those of the authors and do not necessarily represent those of their affiliated organizations, or those of the publisher, the editors and the reviewers. Any product that may be evaluated in this article, or claim that may be made by its manufacturer, is not guaranteed or endorsed by the publisher.
