# Supplementary material for: Auxiliary Metabolic Gene Functions in Pelagic and Benthic Viruses of the Baltic Sea
Source: Front Microbiol. 2022 Jul 7;13:863620. doi: 10.3389/fmicb.2022.863620 (PMC9301287; doi:10.3389/fmicb.2022.863620)
Supplement: Supplementary file 8 [file Data_Sheet_8.PDF]

Supplementary Material

1 SUPPLEMENTARY FIGURES

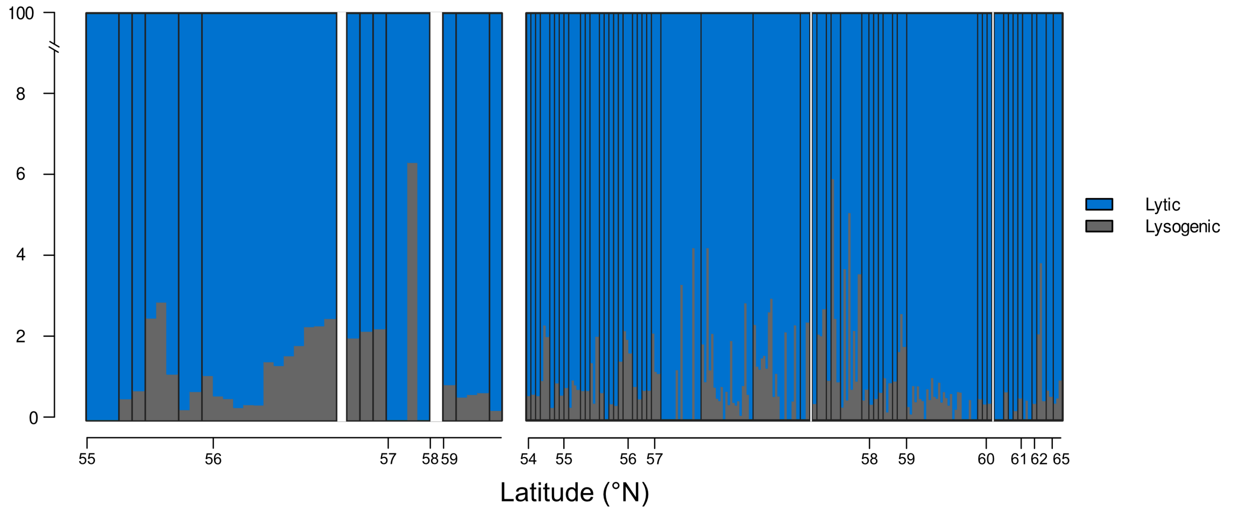

Figure S1. Relative abundance of lytic and lysogenic viral contigs identified by VIBRANT. White space indicate that no viral contigs where found.

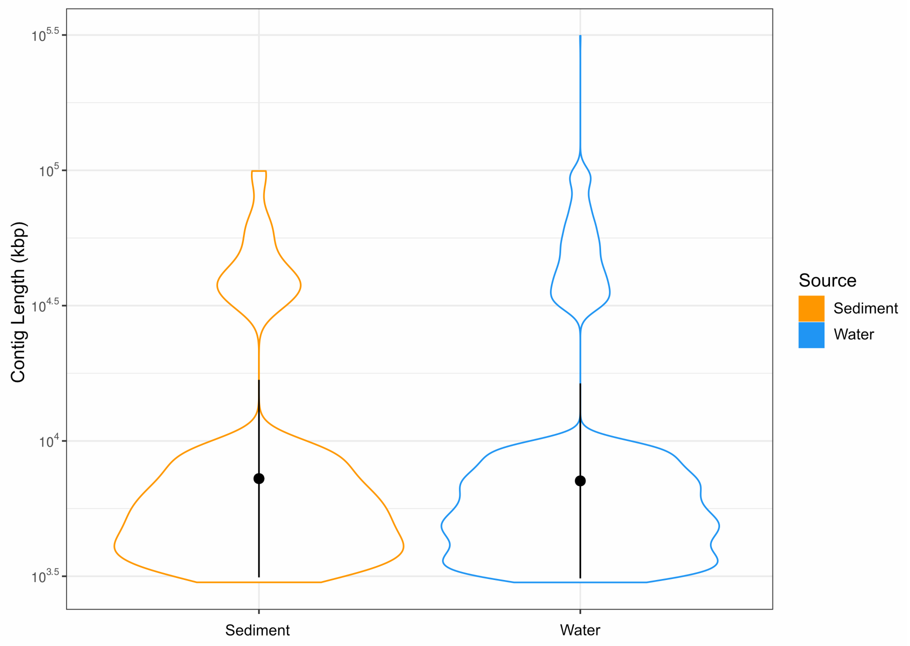

Figure S2. Violin plot of contig length of identified high quality (CheckV score¿ 90%) viral contigs identified in Baltic Sea sediment and water metagenomes. Longest viral contig found in the water column was 314 kbp, while the longest contigs found in the sediment was 100 kbp. The mean viral contig from sediments was 4383 bp and 4493 bp in the water column.

A

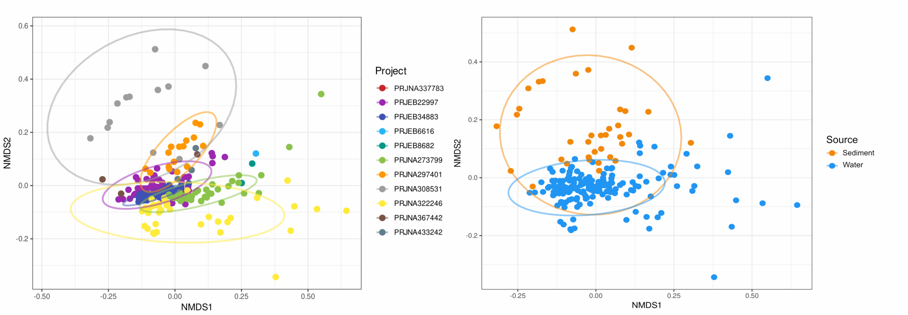

	Df	SumOfSqs	R^2	F	Pr(>F)
Project	10	12.589	0.30723	8.9141	0.001 ***
Residual	201	28.388	0.69277		
Total	211	40.977	1.00000		

Signif. codes: 0 ‘***’ 0.001 ‘**’ 0.01 ‘*’ 0.05 ‘.’ 0.1 ‘ ’ 1

Permutations = 999

B

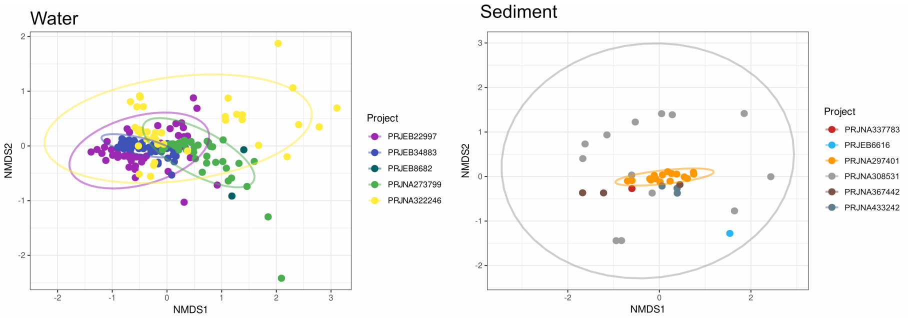

	Df	SumsOfSqs	MeanSqs	F.Model	R^2	Pr(>F)
w.projects	4	6.8631	1.7158	13.198	0.23696	0.001 ***
Residuals	170	22.1001	0.1300	0.76304		
Total	174	28.9631			1.00000	

Signif. codes: 0 ‘***’ 0.001 ‘**’ 0.01 ‘*’ 0.05 ‘.’ 0.1 ‘ ’ 1

Permutations = 999

	Df	SumsOfSqs	MeanSqs	F.Model	R^2	Pr(>F)
s.projects	5	2.4667	0.49334	2.4324	0.28178	0.001 ***
Residuals	31	6.2875	0.20282	0.71822		
Total	36	8.7542	1.00000			

Signif. codes: 0 ‘***’ 0.001 ‘**’ 0.01 ‘*’ 0.05 ‘.’ 0.1 ‘ ’ 1

Permutations = 999

Figure S3. Due to the compositional nature of the metagenomic data used in this study, we assessed the batch effect via Bray-Curtis distance and visualized the results in a NMDS ordination (Supplementary Figure S4). We additionally conducted a PERMANOVA to test whether the different sequencing projects caused a batch effect within our NMDS ordinations. While some project specific clustering could be observed within the ordination, the PERMANOVA showed these effects to be less important ($R^2 = 0.30723$, P <0.001) (Supplementary Figure S4). The batch effect among just water and sediment stations was also insignificant ($R^2 = 0.23696$, p <0.001 and $R^2 = 0.28178$, p <0.001).
